# Brain response in heavy drinkers during cross-commodity alcohol and money discounting with potentially real rewards: A preliminary study

**DOI:** 10.1016/j.dadr.2023.100175

**Published:** 2023-07-06

**Authors:** Elizabeth A. Lungwitz, Mario Dzemidzic, Yitong I. Shen, Martin H. Plawecki, Brandon G. Oberlin

**Affiliations:** a Department of Psychiatry, Indiana University School of Medicine (IUSM); 355 W 16th St, Ste 4800; Indianapolis, IN 46202, USA; b Department of Neurology, IUSM; 355 W 16th St, Ste 4600; Indianapolis, IN 46202, USA; c Department of Radiology and Imaging Sciences, Center for Neuroimaging, IUSM; 355 W 16th St, Ste 4100; Indianapolis, IN 46202, USA; d Department of Psychology, Indiana University Purdue University Indianapolis; 402 N Blackford St, LD124; Indianapolis, IN 46202, USA; e Stark Neurosciences Research Institute, IUSM; 320 W 15th St, Ste 414; Indianapolis, IN 46202 USA

**Keywords:** Alcoholism, Intertemporal choice, Ethanol, Delayed reward discounting, Reinforcer, Ventral striatum

## Abstract

**Background:**

Alcohol use disorder (AUD) is associated with exaggerated preference for immediate rewards, a candidate endophenotype for use disorders. Addiction symptomology is often well-described by the preference for immediate intoxication over other delayed prosocial rewards. We measured brain activation in AUD-implicated regions during a cross-commodity delay discounting (CCD) task with choices for immediate alcohol and delayed money.

**Methods:**

Heavy drinkers (*n*=24) experienced a brief intravenous alcohol infusion prime, regained sobriety, then chose between ‘One Shot’ and delayed money in an adjusting delay CCD task (sober and intoxicated); also during fMRI (sober). Participants also performed a behavioral sensation seeking task and completed self-report inventories of other risk factors. We assessed brain activation to choices representing immediate intoxication versus delayed money rewards in *a priori* regions of interest defined within the framework of Addictions NeuroImaging Assessment.

**Results:**

Activation to CCD choice versus control trials activated paralimbic and ventral frontal cortical regions, including orbital and medial prefrontal cortex, posterior cingulate/retrosplenial cortex, angular and superior frontal gyri. We detected no differences between immediate or delayed choices. Left medial orbitofrontal cortex activation correlated with alcohol-induced wanting for alcohol; females showed greater activation than males. Behavioral sensation seeking correlated with right nucleus accumbens task engagement.

**Conclusions:**

Alcohol decision-making elicited activation in regions governing reward, introspection, and executive decision-making in heavy drinkers, demonstrating the utility of laboratory tasks designed to better model real-world choice. Our findings suggest that the brain processes subserving immediate and delayed choices are mostly overlapping, even with varied commodities.

## Highlights

• Heavy drinkers activate ventral frontal cortex when considering immediate alcohol.
• Orbitofrontal activation correlated with alcohol-primed wanting for alcohol.
• Nucleus accumbens response to alcohol choice correlated with sensation seeking.
• Females show more reward activation than males when considering immediate alcohol.

## Graphical abstract

**Figure:**
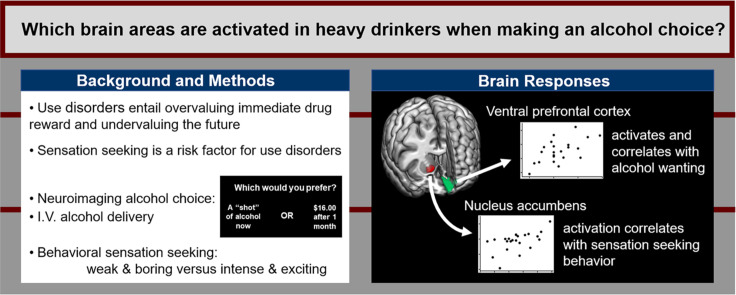

## 1 Introduction

Behavioral impulsivity is the preference for immediate rewards and underweighting of delayed consequences (Logue, 1995). Impaired capacity to delay reward is a candidate endophenotype for addictions and present across multiple drug types (Amlung et al., 2017; Bickel et al., 2014; MacKillop, 2013). Delay discounting (DD) tasks quantify this tendency by pitting the drive for immediate reward against the more adaptive strategy of waiting for larger delayed rewards. Discounting rates are associated positively with alcohol and substance use disorder (AUD, SUD) severity (Amlung et al., 2017), and negatively with successful abstinence (MacKillop and Kahler, 2009; Sheffer et al., 2014; Washio et al., 2011). Steep discounting appears to mark a biological predisposition to addictive disorders, as it is a correlated trait in selectively-bred animal models of AUD (Beckwith and Czachowski, 2014; Oberlin and Grahame, 2009; Wilhelm and Mitchell, 2008), is heritable (Anokhin et al., 2015), has trait-like features (Odum, 2011; Odum, 2011), and confers premorbid risk in humans (Acheson et al., 2011). Combining discounting tasks with neuroimaging techniques permits measuring brain activation governing intertemporal decision-making by illuminating brain activity while salient drug rewards compete against more prosocial future rewards.

Real-world drinking incorporates intertemporal choices that differ by commodity—a point highlighted by recent suggestions to investigate more complex aspects of discounting behavior (Green and Myerson, 2019; Pritschmann et al., 2021), e.g., mixing commodities in discounting tasks. Findings from cross-commodity discounting (CCD) tasks resemble the higher discounting observed when a drug is one of the options under consideration (Bickel et al., 2011; Yoon et al., 2018). CCD tasks are more sensitive than single-commodity DD to drug deprivation (Mitchell, 2004), severity of use (Moody et al., 2017; Naudé et al., 2021; Taylor et al., 2023), and in predicting abstinence (Yoon et al., 2009). CCD tasks are thus uniquely positioned to study the neural processing that occurs during intertemporal decision-making involving drug reward and may provide better ecological validity than traditional DD tasks. The sparse neuroimaging literature using drug CCD tasks implicates the striatum and prefrontal cortex (Wesley et al., 2014), but brain activation during alcohol intertemporal decision-making is still poorly understood.

Using a dimensional approach to psychiatric research, the National Institute of Mental Health launched the Research Domain Criteria project (RDoC; Cuthbert and Insel, 2010) to “identify the fundamental behavioral components” underlying mental disorders for neuroscience research (Cuthbert and Insel, 2013). Extending this rationale to the neurobiology of AUD, Voon et al. (2020) reviewed AUD neuroimaging data using the Addictions Neuroclinical Assessment framework (Kwako et al., 2016) organized around RDoC and addiction domains (Yucel et al., 2019) and neurocircuitry (Koob and Volkow, 2016) implicated in AUD/alcohol misuse. Neuroimaging studies converged on key regions in the default mode, frontoparietal, and salience networks as well as limbic and striatal structures. Any findings in these regions should be examined in light of the well-established risk factors for AUD. Impulsivity, sensation seeking, depression, recent drinking, and response to alcohol are all associated with AUD (Coskunpinar et al., 2013; Gunzerath et al., 2004; Hasin and Grant, 2002; Hasin et al., 2007; King et al., 2021), with alcohol-related activation expected in striatal, ventral frontal reward, and salience network regions based on prior work with alcohol cues (Schacht et al., 2013; Zeng et al., 2021) and high-intensity stimuli (Burnette et al., 2019). Sex effects in AUD manifest in personality (Sannibale and Hall, 2001), gene expression (Ferrer et al., 2020), and brain activation (Smith et al., 2023). While AUD occurs in both males and females (and at increasingly similar rates; Dawson et al., 2015, Keyes et al., 2011), the etiologies may differ by sex, with drug and stress cue-induced activation of striatum in males and vmPFC hypoactivation females predicting subsequent use (Smith et al., 2023). These patterns of neural activation broadly comport with reward-seeking in male AUD (Kuntsche and Müller, 2011) and heightened anxiety in female AUD (Sannibale and Hall, 2001).

Our hypothesis is that when immediate alcohol is a choice under consideration, the brain of a problem drinker engages AUD-relevant regions. Significant activation would identify the regions contributing to the cognitive components underlying preference for immediate intoxication—as distinct from purely habit- or reward-based decision making. We administered a CCD (immediate alcohol-delayed money) task with potentially real rewards during fMRI to quantify engagement of AUD-related brain regions outlined by Voon et al. (2020) during alcohol decision-making. Importantly, participants believed that reward delivery was contingent on choice behavior (“potentially real”, i.e., that some of their selections would be paid out in alcohol and/or money according to choice). We further tested regional brain responses for associations with the response to alcohol, recent drinking, and risk traits (including behavioral sensation seeking).

## 2 Methods

### 2.1 Participants

**Fig. 1:**
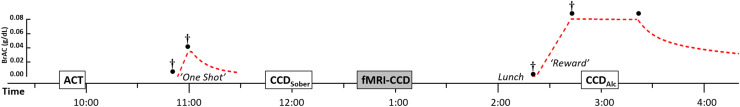
*Study Day Procedures.* Subjects performed the aroma choice task (ACT) and CCD_Sober_ (preceded by brief ‘One Shot’ alcohol infusion), and after returning to 0.00 g/dL BrAC, performed CCD during fMRI (shaded box). Subjects then completed CCD (CCD_Alc_) while maintained at a controlled level of intoxication (*‘Reward’*). Targeted BrAC (y-axis labels) profile is shown in time (dashed red line)—reaching zero at ∼5:30PM. Subjective intoxication ratings were collected pre-infusion and at BrAC peaks (daggers), with BrAC (filled circles) measured concomitantly, and at the end of the clamp. Personality inventories were counterbalanced and administered during sober periods before fMRI (omitted here for clarity).

Thirty-two non-treatment-seeking heavy drinking participants were recruited through community advertisements, gave informed consent prior to study procedures, and were paid in cash ($220) at the end of the study day for participation. Eight subjects provided incomplete data sets resulting in the *n*=24 complete data sets reported here^1^ (Table 1); all participants were a subset of a previously published study (Halcomb et al., 2022). All procedures were approved by the Indiana University Institutional Review Board. Recent drinking history and alcohol problem severity were determined at the in-person interview with the 35-day Timeline Followback (TLFB; Sobell et al., 1986), the Alcohol Use Disorders Identification Test (AUDIT; Saunders et al., 1993), and the Semi-Structured Assessment for the Genetics of Alcoholism (SSAGA; Bucholz et al., 1994). Heavy drinking was defined as AUDIT scores ≥8 and/or exceeding heavy drinking day or week limits prescribed by NIAAA, i.e., exceeding limits of 3 or 4 drinks per day, or 7 or 14 drinks per week for females or males, respectively (Gunzerath et al., 2004; NIAAA 2023). SSAGA revealed lifetime DSM-IV criteria counts exceeding abuse or dependence thresholds (≥3) in 21 participants (88% of the sample). On the study day, subjects reported to the Indiana University Clinical Research Center and completed personality measures, performed discounting tasks outside and in the MRI scanner, and underwent intravenous alcohol infusion (2.2.4 and Figure 1). All tasks and inventories were presented with Eprime 2.0 (Psychology Software Tools, Inc, Sharpsburg, PA) on a laptop computer. Participants were instructed to abstain from alcohol for 48 hours prior to the study. Exclusionary criteria included interest in AUD treatment, a positive urine screen for illicit drugs (except marijuana/THC), nonzero breath alcohol (BrAC) at the interview or study day, history of smell or taste disorders, positive urine pregnancy screen, current use of any psychotropic medication, history or presence of organic brain syndrome, current treatment for psychiatric disorders (including substance use disorder), or major medical disorders that limit behavioral performance. Five participants tested positive for THC on the study day, with the last self-reported use 3.6 ±2.2 days prior; no visible signs of THC intoxication were observed. See Table S3 for details on illicit drug use. Daily nicotine users were offered a nicotine patch during the study to mitigate nicotine withdrawal, with dosing (7-14mg) per manufacturer's recommendations. The nicotine patch was selected as a minimally-invasive nicotine withdrawal method that complied with the campus-wide prohibition of tobacco smoking.

**Table 1:** Subject characteristics (*n*=24).

	Mean (SD)	Range	*n*(%)
Male			13 (54)
Caucasian			13 (54)
African American			9 (38)
Mixed race			2 (8)
Nicotine^a^			17 (71)
Family history positive^b^			14 (58)
Age	32.0 (5.9)	22-43	
Education (years)	13.2 (1.8)	11-18	
Drinks per week^c^	40.5 (24.6)	16.4-116.3	
Drinks per drinking day^c^	8.0 (5.0)	2.6-21.3	
Heavy drinking days per week^c^^,^^d^	3.4 (1.9)	1.2-7.0	
AUDIT^e^	18.1 (6.6)	8-34	
DSM-IV criteria, lifetime^f^	5.1 (2.7)	1-10	

a Daily nicotine use. All nicotine users smoked cigarettes (9.9 ±6.3 per day; range 2-20).

b At least one first-degree relative with probable AUD.

c From the Alcohol Timeline Followback Interview (TLFB).

d ≥4 or 5 drinks on a drinking day for female or male, respectively.

e Alcohol Use Disorders Identification Test.

f Diagnostic and Statistical Manual of Mental Disorders IV, alcohol abuse and dependence endorsements, assessed with the Semi-Structured Assessment for the Genetics of Alcoholism (SSAGA).

### 2.2 Study day procedures

In the morning, participants performed CCD while sober (CCD_Sober_), but after receiving an intravenous infusion representing ‘One Shot’ used as the immediate reward option in the CCD task. Breath alcohol measurement was required to be 0.00 g/dl prior to administering CCD. This initial priming exposure familiarized participants with the intravenous alcohol experience and provided them with the subjective effects of a known unit of intoxication. Participants also performed the behavioral sensation seeking task, the aroma choice task (ACT; Oberlin et al., 2020) and completed personality inventories. Following the CCD_Sober_ task, participants performed an individualized CCD task during fMRI. Afterward, they ate lunch, then were infused with alcohol (individually tailored by CAIS [1.2.2.4] to reach and maintain 0.08 g/dl BrAC) and performed the same adjusting CCD but while held at a constant breath alcohol concentration (CCD_Alc_). The order of the personality inventories was pseudorandomized around the behavioral tasks.

#### 2.2.1 Aroma choice task (ACT)

The ACT quantifies sensation seeking behavior as the relative preference for a mild, safe odorant *‘Standard’* versus a more intense, novel, and variable option that may be aversive *‘Varied’* (Oberlin et al., 2020). The manual sniff bottle version (Oberlin et al., 2021) was used in the present study. The number of “Varied” choices selected divided by the total (40) yields the choice ratio, which ranges from 0 to 1, with larger values indicating a greater degree of behavioral sensation seeking.

#### 2.2.2 Cross commodity discounting (CCD)

Participants chose between the immediate ‘One Shot’ alcohol exposure and delayed money. The immediate reward was always ‘One Shot’, and delayed rewards were $2, $4, $8, and $16; the dollar amounts were selected to bracket the presumed value of a single drink. The starting delay of 30 days was adjusted using an adaptive procedure (modeled after Du et al., 2002). Delayed or immediate choices resulting in doubling or halving, respectively, of the delay for the next trial or, after a preference reversal, the halfway point between the current choice's delay and the previous reversal's delay. Each amount was presented six times, for 24 trials total. Four additional control trials (magnitude discrimination) were randomly included to ensure attentive responding. To counter the strategy of avoiding alcohol choices to hasten release, subjects were instructed that they may not leave before 5:30PM (Oberlin et al., 2021). We titrated delay to avoid fractional—and potentially unintuitive—units of ‘One Shot’ for the immediate reward (Locey et al., 2023).

#### 2.2.3 In-scanner discounting (fMRI-CCD)

To elicit brain activation near indifference points and provoke similar numbers of immediate and delay choices, individualized discounting task versions were created using the indifference points derived in the morning session (CCD_Sober_) and generated biased choices equally above and below the calculated indifference line^2^. fMRI-CCD utilized curve-fitting for modeling indifference: *y*=*y*_0_*exp(*k*A) (Levenberg-Marquardt exponential growth, robust fit; GraphPad Prism 6.0), where A=amount, and *k* and *y*_0_ are fitted parameters.

#### 2.2.4 Intravenous alcohol infusion

Two alcohol infusions were delivered, one in the morning (‘One Shot’; breath alcohol concentration [BrAC] target of 0.035 g/dl in 6 min.) and again in the afternoon (‘Reward’ target of 0.08 g/dl BrAC in 20 min). The ‘One Shot’ served as a reward reference for participants’ CCD decisions, and the ‘Reward’ was ostensibly consequent of alcohol selections (to maintain the belief in contingent alcohol). The ‘Reward’ was standardized to the individualized amount determined by the Computer-assisted Alcohol Infusion System (CAIS; Zimmermann et al., 2008, 2009) to reach and maintain 0.08 g/dl until CCD_Alc_ and ratings completion. Participants believed that rewards were contingent on choices and were told that “some percentage” of alcohol and money choices would be delivered according to selection (obfuscating the precise ratio of reinforcer delivery to mitigate against choosing all money after first selecting the desired amount of alcohol—potentially yielding inaccurate behavioral assessment). We promoted the illusion of contingent reinforcement, as drug commodity discounting can produce different results between hypothetical and potentially real rewards (Green and Lawyer, 2014). Participants’ BrAC <0.02 g/dl was required for release. The perceived contingency between choice and reward was preserved by the delivery of alcohol (infusion), consistent with the alcohol magnitude discrimination trials, and an extra $20 was given to all participants for their monetary choices (with the actual amount/delay obfuscated by “computer selection and rounding”).

#### 2.2.5 Subjective alcohol ratings

Subjects rated alcohol-related effects in a six-item questionnaire with a six-marker scale, at baseline, at the peak of the prime, a second baseline, and at the peak BrAC of the ascent of the Reward infusion. The items “*Right now, I feel as if I've had this many drinks*” ranged from 0-5, and “*I WANT a drink right now*” ranged from “*Strongly disagree*” to “*Strongly agree*”. *“I LIKE how I'm feeling right now”*, “*How INTOXICATED do I feel right now?*”, “*How ANXIOUS do I feel right now?*”, “*Do I feel NUMBNESS or TINGLING in any part of my body?*”, and “*How HIGH do I feel right now?*” were anchored by “*Not at All*” to “*Most Ever*” (Oberlin et al., 2021). Difference scores were calculated by subtracting the baseline ratings from the peak ratings at both time points.

#### 2.2.6 Subjective alcohol value

Following all procedures and just prior to release, participants were asked, *“In the places you would normally buy a shot or drink, how much does it cost?”* to determine subjective price points for alcohol.

#### 2.2.7 Personality self-report

During periods between tasks and while sober, personality assessments of impulsivity were collected. Impulsivity, sensation seeking, and depression were assessed with the shortened UPPS-P Impulsive Behavior Scale (sUPPS-P; Cyders et al., 2014), the Arnett Inventory of Sensation Seeking (AISS; Arnett, 1994), and the Center for Epidemiologic Studies Depression Scale (CES-D; Radloff, 1977), respectively; see Table S2. The order of the tests was pseudorandomized between subjects.

#### 2.2.8 fMRI image acquisition

Imaging was performed on a Siemens 3T Prisma (Erlangen, Germany) MRI scanner with a 32-channel head coil array. fMRI data were collected with a 7 minute and 29 second long scan using a multiband (MB) blood oxygenation level dependent (BOLD) contrast sensitive sequence (Xu et al., 2015) with the following parameters: 546 BOLD volumes, gradient-echo echo-planar imaging (EPI), MB slice acceleration factor= 4, TR/TE= 810/29ms, flip angle= 56°, 2.5​ × ​2.5 × 2.5 ​mm^3^ voxels, field-of-view: 220 × 220 mm, 48 axial slices. The BOLD imaging was preceded by two short (16s) spin echo field mapping scans (TR/TE= 1370ms/51.6ms, 5 A-P and 5 P-A phase direction volumes) with the same coverage, voxel size, and slice acceleration as the BOLD acquisition. At the start of the imaging session, participants underwent a T1-weighted anatomical MRI with whole brain coverage using a 3D Magnetization Prepared Rapid Gradient Echo (MPRAGE) sequence (5 minutes and 12 seconds duration, 176 sagittal slices, 1.05 × 1.05​ × ​1.2 ​mm^3^ voxels, GRAPPA R ​= ​2 acceleration) per the Alzheimer's Disease Neuroimaging Initiative (ADNI-2) imaging protocol. See *Supplemental Information* for detailed fMRI preprocessing description.

#### 2.2.9 fMRI data

Intertemporal choice and control trials utilized an event-related fMRI design with a mean intertrial interval of 11 seconds (see Figure S1 for trial design). The primary contrast of interest in SPM12 models assessed responses of choice relative to control trials; [Choice > Control]. Individual-level [Choice > Control], [Immediate > Delay], and [Delay > Immediate] contrasts were also created. The immediate and delay trials were those from actual choices made, with a minimum requirement of three selections of a given choice type. Using the MarsBar toolbox (Brett et al., 2002), we then extracted mean contrast values from 22 *a priori* regions of interests (ROIs) identified as key brain regions for AUD (Voon et al., 2020). The exploratory whole-brain analysis utilized a group-level ANOVA to identify significant clusters from [Choice > Control]. Significant clusters were then extracted for each participant as in the *a priori* ROI analysis.

### 2.3 Regions of interest

**Fig. 2:**
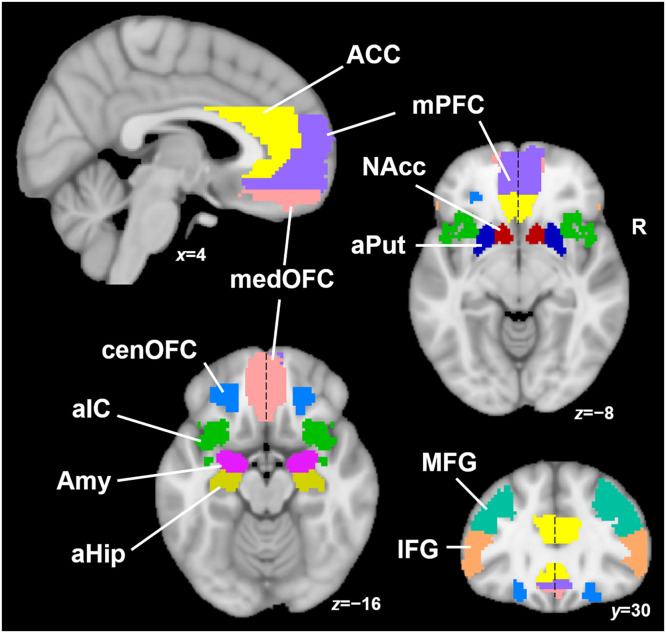
*Regions of Interest.* Spatial boundaries of key brain areas implicated in AUD. ACC (light yellow) anterior cingulate cortex; mPFC (violet) medial prefrontal cortex; medOFC (pink) medial orbitofrontal cortex; cenOFC (light blue) central orbitofrontal cortex; aIC (green) anterior insular cortex; Amy (purple) amygdala; aHip (dark yellow) anterior hippocampus; NAcc (red) nucleus accumbens; aPut (dark blue) anterior putamen; MFG (turquoise) middle frontal gyrus; IFG (orange) inferior frontal gyrus. Note that midline ROIs are split into left and right parts, indicated by dashed black line.

Voon et al. (2020) outlined AUD-relevant regions that included twenty-two ROIs (eleven regions per hemisphere) including nucleus accumbens (NAcc), anterior putamen (aPut), amygdala (Amy), anterior hippocampus (aHip), central and medial orbitofrontal cortex (cenOFC and medOFC), medial prefrontal cortex (mPFC), anterior cingulate cortex (ACC), anterior insular cortex (aIC), inferior and middle frontal gyri (IFG and MFG) as illustrated in Fig. 2. *Supplemental Information* provides more detail on the rationale and spatial locations and boundaries for each ROI; ROIs provided in *Supplemental Files* in .nii format.

### 2.4 Analyses

Analyses of fMRI data were conducted by 1) *a priori* ROIs, then 2) whole brain, to ensure important results outside *a priori* regions were not overlooked. Both analyses evaluated main effects of activation, with significant results tested for effects of important AUD-related factors. Effects of sex were separately tested in regions showing significant activation to CCD choice. Analyses performed outside SPM used SPSS Statistics 27 (IBM Corp., Armonk, NY, USA) with alpha set to .05, with a false discovery rate (B-H FDR; Benjamini and Hochberg, 1995) of 5% preserved for multiple comparisons.

#### 2.4.1 Analyses: alcohol exposure and effects

Measured BrAC values were tested against target values with one sample *t*-tests. Paired *t*-tests evaluated alcohol effects on CCD behavior, with natural-log transformation of area under the curve to normalize data. Difference scores in subjective alcohol ratings were assessed with one sample *t*-tests against zero.

#### 2.4.2 Analyses: fMRI ROIs

Average parameter estimates were extracted for each *a priori* ROI in the [Choice > Control] contrast and evaluated with one-sample two tailed *t*-test against zero. Any region showing significant activation was tested for differences by choice type (mean parameter estimates for region during specified choice, paired-*t*) and correlation with subjective response to alcohol and effects of recent drinking, AUD severity, and personality risk factors. We tested for effects of recent THC use with independent *t*-tests.

#### 2.4.3 Analyses: AUD risk factors

Risk factors (drinks/week, heavy drinking days/week, drinks/drinking day, lifetime DSM-4 criteria, AUDIT, CESD, and AISS and SUPPS-P subscales [13 factors] were dimensionally reduced with principal component analysis to yield two components that represented drinking problems/impulsive urgency/depression and alcohol consumption; see Table S1).

#### 2.4.4 Analyses: sensation seeking

The neural correlates of behavioral sensation seeking (ACT task) were tested for correlations with activation in reward-related ROIs (nucleus accumbens, and central and medial OFC: six regions).

#### 2.4.5 Analyses: fMRI whole brain

Whole-brain voxel-wise exploratory analyses of the [Choice > Control] contrast identified activation foci that satisfied familywise error (FWE) correction for multiple comparisons (cluster-level *p*_FWE_<.001 at a cluster-forming threshold *p*_uncorr_<.001); covaried for age and sex. To ensure robustness, we additionally required each cluster's primary peak to exceed voxel-level corrected significance level *p*_FWE_<.05. Mean parameter estimates were then extracted from these significant clusters and assessed for correlation with alcohol response and risk factors. Similar to the *a priori* ROI analysis, [Immediate > Delay] and [Delay > Immediate] contrasts were also tested in SPM12.

## 3 Results

### 3.1 Alcohol exposure and CCD

The peak of the clamp ascent and maintenance closely approximated the target (*p*s>.11), means 0.084 ±.013 0.082 ±0.012, respectively while the morning priming dose (“One Shot”) exceeded the target of 0.035 g/dl, *t*(23)=3.60, *p*=.002, mean 0.041 ±0.009 g/dl^3^. No effect of alcohol intoxication on CCD behavior was detected (CCD_Sober_ versus CCD_Alc_), *p*=.44.

### 3.2 Subjective alcohol ratings

The priming dose elevated the perceived number of drinks, intoxication, numbness, high, and decreased anxiety, *t*s(23)>2.48, *p*s<.022, but did not change wanting or liking, *p*s>.15. The peak of the ramp produced a similar pattern, with increased ratings of drinks, intoxication, numbness, and high, *t*s>3.31, *p*s<.004, but no change in anxiety, wanting, or liking, *p*s>.03 (note B-H FDR sets alphas <.05). Comparing the baseline ratings, to detect potential lingering effects of the priming dose, revealed no differences, *p*s>.11).

### 3.3 Subjective alcohol value

Participants reported paying an average of $3.79 ±2.48 for typical drinks (median $4.00). Of the 20 participants reporting their typical drinking environment, *n*=17 drank primarily at home.

### 3.4 CCD activation in a priori ROIs

**Fig. 3:**
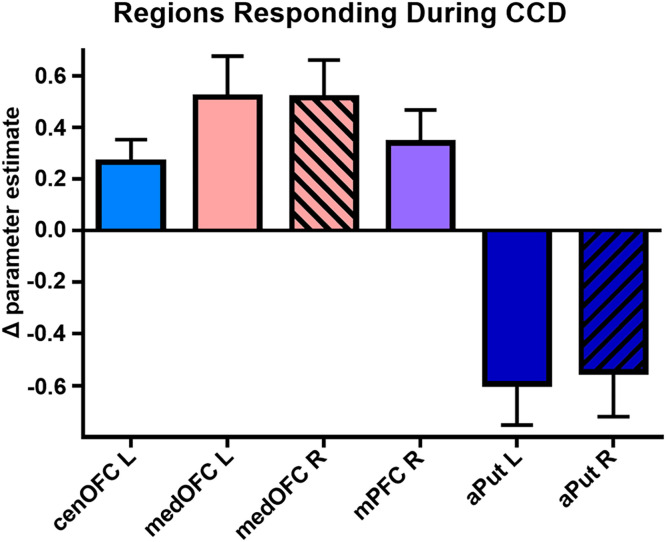
*Responses in AUD-relevant regions.* Ventral frontal and medial limbic regions showed a positive response during CCD choice relative to control (bilateral results shown with the right hemisphere ROI diagonally shaded). Reduced activation during choice trials was detected in the anterior striatum. Means ±SEM from [Choice > Control]. cenOFC = central orbitofrontal cortex; medOFC = medial orbitofrontal cortex; mPFC = medial prefrontal cortex; aPut = anterior putamen.

A greater response during CCD choice relative to control trials was detected in the left central OFC, bilateral medial OFC, and right medial PFC, while a reduced response was detected in the bilateral anterior putamen, *t*s(23)>2.69, *p*s<.014 (B-H FDR *p*<.05); Fig. 3. No difference was detected when immediate choice was compared directly to delayed choice in these regions, *p*s>.24. No effect of THC was detected (*p*s_uncorr_>.007, not meeting B-H correction threshold).

### 3.5 Alcohol risk factors and CCD activation

**Fig. 4:**
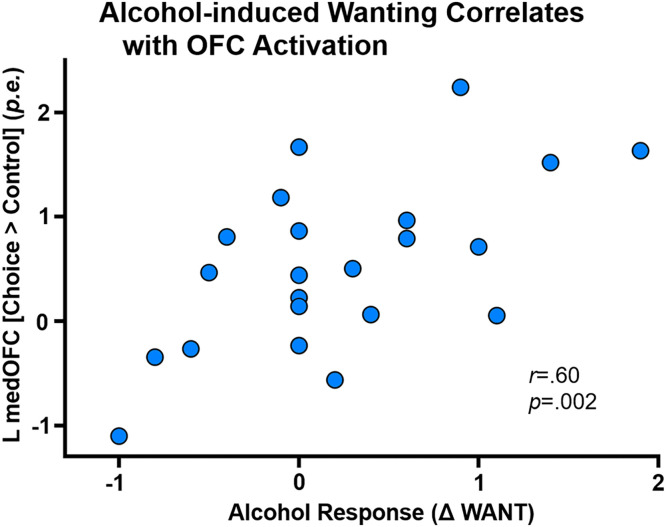
*Wanting induction and orbitofrontal activation.* The left medial orbitofrontal cortex activation during intertemporal alcohol-money choice correlated with the change in WANT (pre-infusion versus peak BrAC from the intravenous alcohol priming dose. *p.e.* = parameter estimate difference in the choice and control conditions.

Alcohol response and risk factors (encompassing recent drinking, AUD severity, and self-reported depression, sensation seeking, and impulsivity) were tested for correlation in *a priori* regions showing significant differences; only the correlation between the left medial OFC and Wanting during the priming dose met the corrected significance threshold, *r*(22)=.60, *p*=.002; Fig. 4.

### 3.6 Behavioral sensation seeking and CCD activation

**Fig. 5:**
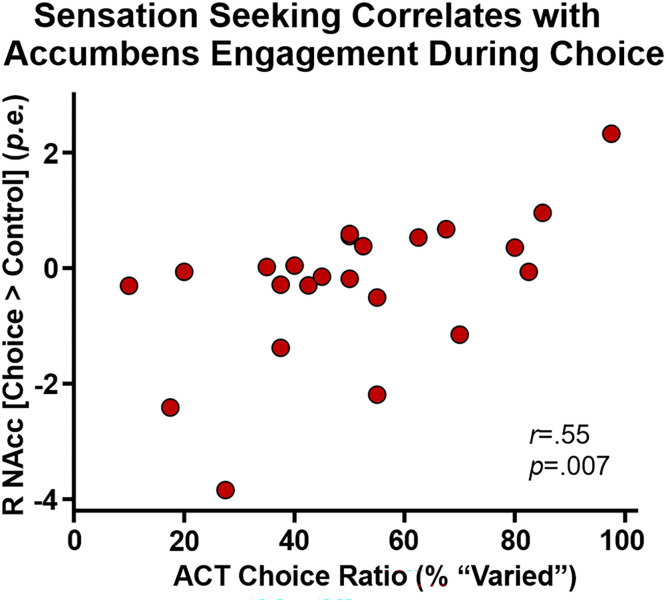
*Behavioral sensation seeking and reward response.* The right nucleus accumbens response during intertemporal alcohol:money choice correlated with the preference for high intensity/novelty/risky olfactory stimuli. *p.e.* = parameter estimate difference in the choice and control conditions.

A greater preference for more intense stimuli was detected in participants with greater right nucleus accumbens response during alcohol intertemporal choice, *r*(21)=.55, *p*=.007; Fig. 5. ACT did not correlate with CCD in either CCD_Sober_, CCD_Alc_, or the pre-alcohol versus post-alcohol change in CCD, i.e., delta CCD (*p*s>.6).

### 3.7 Sex effects

Effects of sex were assessed in the six *a priori* ROIs that showed a significant response to CCD choice. The left medial OFC produced a larger effect in females compared to males, *t*(22)=2.25, *p*=.035; means 0.88 ±0.74 and 0.22 ±0.70, respectively. No effects of sex were detected in CCD_Sober_ or CCD_Alc_, subjective alcohol ratings in either the prime or the ramp, *p*s>.11, in the PCA components representing alcohol risk factors, *p*s>.42, or in ACT choice ratio, *p*=.88. The sexes did not differ by age (*p*=.21) or nicotine use (Chi-squared test, *p*=.66).

### 3.8 CCD whole-brain results

**Fig. 6:**
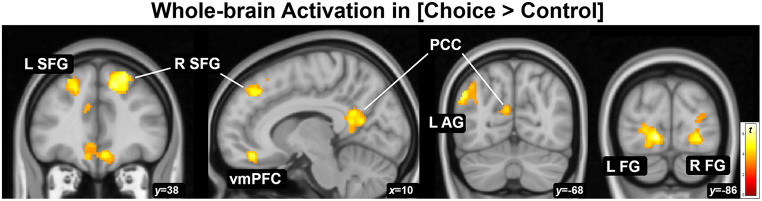
*Whole-brain assessment of activation during choice.* Activation while considering immediate intoxication versus delayed money compared to control (magnitude discrimination), i.e., the [Choice > Control] contrast, produced seven clusters. Clusters meeting corrected significance *p*_FWE_<.05 containing peaks *p*_FWE_<.05 are displayed at *p*_uncorr_<.001; L and R SFG = left and right superior frontal gyrus, vmPFC = ventromedial prefrontal cortex, PCC = posterior cingulate (and retrosplenial) cortex, L AG = left angular gyrus, L and R FG = left and right fusiform gyri.

Exploratory analyses revealed clusters of activation in choice trials [Choice > Control] in the midline default mode and frontoparietal networks and visual areas, i.e., posterior cingulate/retrosplenial cortices (PCC), ventromedial prefrontal cortex (vmPFC), left angular gyrus (AG), bilateral superior frontal gyrus (SFG), and bilateral fusiform gyrus (FG); Fig. 6. The opposite contrast, [Control > Choice] produced activation in the left supramarginal gyus, middle temporal gyrus, and cerebellum; see Table 2 for results. Neither [Immediate > Delay] nor [Delay > Immediate] produced significant results; neither did age nor sex produce significant results.

**Table 2:** Whole-brain voxelwise results for CCD.

			MNI coordinate (mm, peak)		
Region	Cluster size^a^	Peak *Z*^b^	*x*	*y*	*z*
*[Choice ≥ Control]*					
Superior frontal gyrus R	809	6.02	22	36	48
Fusiform gyrus L	534	5.49	−14	−86	−4
Fusiform gyrus R	301	5.19	26	−84	−6
Angular gyrus L	295	5.04	−50	−68	34
Ventromedial prefrontal cortex	359	5.03	0	26	−8
Posterior cingulate/retrosplenial cortex	1116	4.93	6	−50	14
Superior frontal gyrus L	1206	4.92	−24	34	46
					
*[Control ≥ Choice]*					
Supramarginal/postcentral gyrus L	2452	5.46	−56	−24	30
Cerebellum R	212	5.33	44	−54	−28
Middle temporal gyrus L	513	5.28	−54	−52	0

a Number of voxels. All clusters exceed cluster-level significance *p*_FWE_<.001.

b All peaks equivalent to *p*_FWE_<.05. *MNI* Montreal Neurological Institute; *R* right *L* left.

### 3.9 Correlations with CCD activation

Alcohol response and risk factors were tested for correlation with significant clusters in the [Choice > Control] contrast. Only the correlation between left fusiform gyrus activation and ‘number of drinks’ during the priming dose met the corrected significance threshold, *r*(22)=.62, *p*=.001.

### 3.10 THC, behavior, and risk factors

Participants testing positive for THC on the study day (*n*=5) were tested against *n*=19 THC-negative participants for differences in CCD (sober or intoxicated), behavioral sensation seeking, and alcohol risk factors; no effects were detected (*p*s>.32).

#### 3.10.1 Nicotine patch effects

To test for potential novelty effects of the nicotine patch, tobacco smokers who used it (*n*=13) were compared to those who refused (*n*=4) for differences in CCD; no effects were detected (*t*-test; CCD_Sober_ *p*=.90, CCD_Alc_ *p*=.45).

## 4 Discussion

In what we believe is the first CCD-fMRI study utilizing alcohol and potentially real rewards, we present a paradigm designed to model the consideration of drinking alcohol versus delayed monetary rewards. Heavy drinkers demonstrated brain activation in areas largely in common with other discounting work—especially ventral frontal reward- and value-related areas. Notably, orbitofrontal activation correlated with alcohol-induced wanting for more alcohol. Task engagement of the nucleus accumbens during alcohol CCD decision-making correlated with higher behavioral sensation-seeking, suggesting a site of interaction between the proclivity for seeking high intensity stimuli and alcohol-related decision making. In whole-brain analyses, we also detected midline default mode involvement during alcohol decision-making. Unexpectedly, we detected a larger effect in the left medial OFC in females compared to males. No sex effects in self-report or behavior were found, however. These findings demonstrate that laboratory models of SUD-related decision making (immediate intoxication versus other delayed rewards) show considerable promise in measuring brain processes of interest for AUD.

CCD assessment presents unique challenges compared to standard DD tasks. Unlike choices of a strictly intertemporal nature, CCD tasks implemented with current users’ drug of choice potentially elicit conditioned responses analogous to cue reactivity studies—that is, the thought of drug-taking necessarily becomes part of the decision process. Therefore, CCD tasks offering intoxication potentially engage brain areas subserving both drug taking *and* intertemporal decision making. The integration of disparate reinforcer types presumably involves common value signal encoding—a proposed function of the OFC/vmPFC (Ballesta et al., 2020; Hare et al., 2009; Westbrook et al., 2019). Neuroimaging studies find overlap in the vmPFC/OFC in cue reactivity (Zeng et al., 2021) and monetary DD (Schüller et al., 2019), and in manipulations to attenuate both (Oberlin et al., 2020). We find left medial OFC activation to the simultaneous processes of intertemporal decision-making and consideration of alcohol intoxication. The role of the medial OFC as a site for integrating disparate drug-related cognitive processes is further supported by the positive correlation between alcohol-induced increases in drug wanting and activation during CCD decision-making.

The frontoparietal network subserves executive function, e.g., planning, attention, and control (Dosenbach et al., 2008; Fischer et al., 2021; Niendam et al., 2012) and is, unsurprisingly, engaged by intertemporal decision-making tasks (Blain et al., 2016; McClure et al., 2007; McClure et al., 2004). The attractive idea that executive training enhancement will enhance SUD remission (Bickel et al., 2015) is tempered by the observation that frontoparietal activity, insofar as it governs executive function, can be co-opted for maladaptive decision-making, such as the decision to use illicit drugs (Bedi et al., 2015). Our whole-brain analysis revealed both frontoparietal and default mode involvement during alcohol CCD decision-making. This suggests that the executive frontoparietal network accesses the introspective default mode network coincident with ventral frontal value signals for a final action computation. High temporal resolution connectivity studies would be required to address this explanation convincingly.

We found putamen deactivation during choice, which may seem unintuitive. However, the results from Wesley et al. (2014) suggests that putamen activation may mark differential sensitivity to the temporal availability of money governed by task contingencies. When money is available and chosen now, (Money-Cocaine task) chronic cocaine users (CCU) respond more strongly than controls, but when delayed money is available and chosen, (Cocaine-Money task; most analogous to our task) CCU's putamen response is less than controls. This pattern resembles the current findings in that heavy drinkers’ putamen is underactive in choices involving delayed money, relative to control trials.

Sensation seeking is characterized by the drive for salient and intense stimuli (Arnett, 1994; Whiteside and Lynam, 2001; Zuckerman, 1990); similarly, SUDs comprise consuming brief intense reinforcers. The correlation between drug taking and sensation seeking is well established (Arnett, 1994; Oberlin et al., 2020; Silveira et al., 2019) and should be unsurprising as AUD entails avidity for intense stimuli. Prior work implicates limbic and striatal reward sensitivity to drug cues in high sensation seekers (Burnette et al., 2019), and similarly with non-drug high intensity stimuli (Bjork et al., 2008; Joseph et al., 2009). We extend these findings to drug-specific intertemporal decision-making using behavioral choice for actual sensory experiences (the ACT).

As the current sample size is modest, this report should be considered preliminary. A shortcoming in the CCD task used was the limited delay adjustment range, which could be expanded for future studies to potentially include more patient participants. Exclusion of other comorbidities, especially substance use disorder, may have restricted our sample to higher-functioning participants. The lack of a control group could be viewed as a limitation, but this approach was taken deliberately, as the expected valuation differences for alcohol between risky drinkers and social drinkers would potentially confound CCD decision-making and activation. While this study targeted *a priori* regions previously identified as important in AUD, it was partly exploratory in that we aimed to identify which regions were implicated in CCD decision making but did not specify which we believed were most critical. While some past work suggests that immediate versus delayed choice is governed by different brain regions (McClure et al., 2004), other work found no evidence of such separate systems (Glimcher et al., 2007; Kable and Glimcher, 2010)—consistent with our recent report suggesting largely overlapping activation by choice type (Butcher et al., 2021).

This report adds support to converging evidence of the critical role for the vmPFC/OFC in valuation and decision-making as it relates to addictive disorders. The novel demonstration of relating behavioral sensation seeking and accumbens engagement during alcohol choice importantly extends the sensation seeking literature with behavioral testing and actual stimuli. Elucidating brain responses in addictive disorders with behavioral tasks targeting ecologically relevant outcomes will meaningfully enhance our understanding of brain dysfunction and should drive progress in AUD/SUD treatment.

## 5 Declarations

### 5.1 Role of funding source

Effort on this manuscript was supported by the National Institute on Alcohol Abuse and Alcoholism, grants R00 AA023296 (BGO), P60 AA07611 (PI: David Kareken) through the Indiana Alcohol Research Center (IARC), and internal funding from the Department of Psychiatry at the Indiana University School of Medicine. Clinical support was funded in part through the Indiana Clinical and Translational Sciences Institute Clinical Research Center, UL1 TR001108, NIH, National Center for Advancing Translational Sciences, Clinical and Translational Sciences Award (PI: Anantha Shekhar). The funders had no role in the conduct of the study, manuscript preparation, or the decision to submit for publication. The views expressed in this manuscript are those of the authors and do not necessarily reflect the position or policy of the funders.

## CRediT authorship contribution statement

**Elizabeth A. Lungwitz:** Formal analysis, Visualization, Writing – original draft, Writing – review & editing. **Mario Dzemidzic:** Data curation, Methodology, Resources, Software, Writing – review & editing. **Yitong I. Shen:** Data curation, Writing – review & editing. **Martin H. Plawecki:** Resources, Software, Writing – review & editing. **Brandon G. Oberlin:** Conceptualization, Funding acquisition, Methodology, Project administration, Resources, Supervision, Software, Writing – original draft, Writing – review & editing.

## Declaration of Competing Interest

There are no financial or personal interests or beliefs that could affect the objectivity of this manuscript.

## Appendix Supplementary materials

Image, application 1

Image, application 2

Image, application 3

Image, application 4

## Footnotes

1 Of the eight excluded participants, one showed zero discounting in the CCD_Sober_ task, making the MRI task non-parameterized (thus omitted); one had a previously undisclosed contraindication for MRI; four reported strategically avoiding alcohol choices with the intent of earlier release; one showed less than 90% correct on control trials, and one did not meet the minimum threshold of three selections of one choice type during fMRI (delay or immediate). One participants’ Aroma Choice Task data were discarded due to misunderstanding the instructions.

2 If participants selected all delayed choices for a particular amount in CCD_Sober_ (making the task unable to generate sufficiently long delays in 6 trials for preference reversal), an indifference point of 1.5x the maximum was imputed for that delay and used in subsequent curve-fitting to prepare the fMRI task; this was implemented after the first occurrence and required for two later participants.

3 The deviation from prime versus clamp target was attributable to technical differences in how BrAC feedback is used to adjust the model and subsequently alter the participant's BrAC.
