# Supplementary material for: Brain response in heavy drinkers during cross-commodity alcohol and money discounting with potentially real rewards: A preliminary study
Source: Drug Alcohol Depend Rep. 2023 Jul 6;8:100175. doi: 10.1016/j.dadr.2023.100175 (PMC10518510; doi:10.1016/j.dadr.2023.100175)
Supplement: Supplementary file 1 [file mmc1.docx]

Figure S1. Example illustration of visual elements displayed for discounting tasks for both the adjusting task and the fMRI version. Time constraints shown only apply to the fMRI task version. ISI = interstimulus interval.
