# Supplementary material for: Brain response in heavy drinkers during cross-commodity alcohol and money discounting with potentially real rewards: A preliminary study
Source: Drug Alcohol Depend Rep. 2023 Jul 6;8:100175. doi: 10.1016/j.dadr.2023.100175 (PMC10518510; doi:10.1016/j.dadr.2023.100175)
Supplement: Supplementary file 2 [file mmc2.docx]

**Supplemental Information**

*Intravenous alcohol infusion.* Alcohol was systematically infused to attain a standardized intoxication level (brain exposure) using a physiologically based pharmacokinetic model of alcohol metabolism. This integrated hardware/software system (computerized alcohol infusion system; CAIS), incorporates the participants’ age, sex, and weight to generate individualized infusion profiles (O'Connor, Morzorati et al. 1998, Plawecki, Decarlo et al. 2007). To ensure precise targeting and error correction, breath alcohol (BrAC) levels were assessed during infusion and provided to the CAIS system for real-time refinement.

*Region of interest definitions.* Voon et al. (2020) outlined three functional domains and ten brain regions implicated in key AUD phenotypes from meta-analytic and task-based neuroimaging studies. Using the cited manuscripts and refining those descriptions with published atlases and parcellations, we defined the ROIs according to the following procedures. Note that the double quotes indicate the region’s name as published in its original source, with our naming convention in square brackets. *ROIs:* Limbic and subcortical region boundaries were defined using the connectivity gradients proposed by the Tian *et al.* (2020) parcellation (Scale II, 3T): nucleus accumbens [NAcc] from “NAc-shell” (Kuhn and Gallinat 2011); anterior putamen [aPut] “aPUT” (Norman, Pulido et al. 2011, Sjoerds, de Wit et al. 2013); combined medial and lateral amygdala [Amy] “mAMY” and “lAMY” (Wiers, Stelzel et al. 2015, Gowin, Vatsalya et al. 2016, Nikolova, Knodt et al. 2016); anterior hippocampus [aHip] “aHIP” (Chase, Eickhoff et al. 2011, Engelmann, Versace et al. 2012). Orbitofrontal ROIs were derived from dopaminergic resting-state connectivity maps defined by Kahnt and Tobler (2017). “Central” and “medial” parcels are mid-lateral and midline, respectively [cenOFC] and [medOFC]—the medial parcel extends dorsally into medial PFC, so is exclusively masked by the mPFC ROI. [mPFC] “ventromedial prefrontal cortex” defined by Sjoerds *et al.* (2013) was derived from the automated anatomical labeling atlas (AAL3; Rolls, Huang et al. 2020). Anterior cingulate cortex [ACC] included AAL3 regions “ACC_sup”, “ACC_pre”, and “ACC_sub” (Beck, Schlagenhauf et al. 2009, Engelmann, Versace et al. 2012, Seo, Lacadie et al. 2013, Luijten, Machielsen et al. 2014). The anterior insular cortex [aIC] was defined by functionally-defined subregions from an online repository (neurovault.org; Gorgolewski, Varoquaux et al. 2015), dorsal and ventral anterior subregions thresholded at 80%. Lateral prefrontal cortex was defined by FSL’s Harvard-Oxford cortical atlas as inferior frontal gyrus [IFG] “Frontal_Mid” and middle frontal gyrus [MFG] “Frontal_Sup” (Chase, Eickhoff et al. 2011, Engelmann, Versace et al. 2012). All regions were non-overlapping and composed of contiguous voxels.

*fMRI image preprocessing.* BOLD images were preprocessed using FSL (FMRIB’s Software Library (Jenkinson, Beckmann et al. 2012). Specifically, we included BOLD volume distortion correction utilizing spin echo field mapping scans as implemented in *topup/applytopup*, motion correction with *mcflirt*, non-brain removal with *bet*, spatial smoothing with a 6.0 mm full width at half maximum Gaussian kernel, and mean intensity normalization of volumes at each timepoint. Linear registration to high resolution structural and standard space images was carried out using *flirt* and was followed by *fnirt* nonlinear registration. Following recommendations for robust preprocessing (Eklund, Knutsson et al. 2019), the preprocessed data were presented to FSL’s *MELODIC* to generate filtered data for independent component analysis (ICA)-based denoising with ICA-AROMA (Pruim, Mennes et al. 2015). The denoised functional data were then projected in the standard Montreal Neurological Institute (MNI) space and interpolated to 2 mm isotropic voxels. Subsequent statistical analyses in SPM12 were performed on the denoised data.

*fMRI data.* Individual-level responses to each trial were modeled in SPM12 using canonical hemodynamic response function and FAST autocorrelation modeling (Olszowy, Aston et al. 2019) appropriate for short TR data. Trials were modeled to capture the choice consideration and decision-making period—that is, the period during viewing choice options, but immediately preceding the response—with the onset 400 ms after choice presentation (allowing for semantic comprehension; Hagoort, Hald et al. 2004) and duration ending 50 ms before response (to minimize motor signal; Pfefferbaum, Ford et al. 1985).

**References**

Beck, A., F. Schlagenhauf, T. Wustenberg, J. Hein, T. Kienast, T. Kahnt, K. Schmack, C. Hagele, B. Knutson, A. Heinz and J. Wrase (2009). "Ventral striatal activation during reward anticipation correlates with impulsivity in alcoholics." Biol Psychiatry **66**(8): 734-742.

Chase, H. W., S. B. Eickhoff, A. R. Laird and L. Hogarth (2011). "The neural basis of drug stimulus processing and craving: an activation likelihood estimation meta-analysis." Biol Psychiatry **70**(8): 785-793.

Eklund, A., H. Knutsson and T. E. Nichols (2019). "Cluster failure revisited: Impact of first level design and physiological noise on cluster false positive rates." Hum Brain Mapp **40**(7): 2017-2032.

Engelmann, J. M., F. Versace, J. D. Robinson, J. A. Minnix, C. Y. Lam, Y. Cui, V. L. Brown and P. M. Cinciripini (2012). "Neural substrates of smoking cue reactivity: a meta-analysis of fMRI studies." Neuroimage **60**(1): 252-262.

Gorgolewski, K. J., G. Varoquaux, G. Rivera, Y. Schwarz, S. S. Ghosh, C. Maumet, V. V. Sochat, T. E. Nichols, R. A. Poldrack, J. B. Poline, T. Yarkoni and D. S. Margulies (2015). "NeuroVault.org: a web-based repository for collecting and sharing unthresholded statistical maps of the human brain." Front Neuroinform **9**: 8.

Gowin, J. L., V. Vatsalya, J. G. Westman, M. L. Schwandt, S. Bartlett, M. Heilig, R. Momenan and V. A. Ramchandani (2016). "The Effect of Varenicline on the Neural Processing of Fearful Faces and the Subjective Effects of Alcohol in Heavy Drinkers." Alcohol Clin Exp Res **40**(5): 979-987.

Hagoort, P., L. Hald, M. Bastiaansen and K. M. Petersson (2004). "Integration of word meaning and world knowledge in language comprehension." Science **304**(5669): 438-441.

Jenkinson, M., C. F. Beckmann, T. E. Behrens, M. W. Woolrich and S. M. Smith (2012). "Fsl." Neuroimage **62**(2): 782-790.

Kahnt, T. and P. N. Tobler (2017). "Dopamine Modulates the Functional Organization of the Orbitofrontal Cortex." J Neurosci **37**(6): 1493-1504.

Kuhn, S. and J. Gallinat (2011). "Common biology of craving across legal and illegal drugs - a quantitative meta-analysis of cue-reactivity brain response." Eur J Neurosci **33**(7): 1318-1326.

Luijten, M., M. W. J. Machielsen, D. J. Veltman, R. Hester, L. de Haan and I. H. A. Franken (2014). "Systematic review of ERP and fMRI studies investigating inhibitory control and error processing in people with substance dependence and behavioural addictions." Journal of psychiatry & neuroscience.

Nikolova, Y. S., A. R. Knodt, S. R. Radtke and A. R. Hariri (2016). "Divergent responses of the amygdala and ventral striatum predict stress-related problem drinking in young adults: possible differential markers of affective and impulsive pathways of risk for alcohol use disorder." Mol Psychiatry **21**(3): 348-356.

Norman, A. L., C. Pulido, L. M. Squeglia, A. D. Spadoni, M. P. Paulus and S. F. Tapert (2011). "Neural activation during inhibition predicts initiation of substance use in adolescence." Drug Alcohol Depend **119**(3): 216-223.

O'Connor, S., S. Morzorati, J. Christian and T. K. Li (1998). "Clamping breath alcohol concentration reduces experimental variance: application to the study of acute tolerance to alcohol and alcohol elimination rate." Alcohol Clin Exp Res **22**(1): 202-210.

Olszowy, W., J. Aston, C. Rua and G. B. Williams (2019). "Accurate autocorrelation modeling substantially improves fMRI reliability." Nat Commun **10**(1): 1220.

Pfefferbaum, A., J. M. Ford, B. J. Weller and B. S. Kopell (1985). "ERPs to response production and inhibition." Electroencephalogr Clin Neurophysiol **60**(5): 423-434.

Plawecki, M. H., R. Decarlo, V. A. Ramchandani and S. O'Connor (2007). "Improved Transformation of Morphometric Measurements for a Priori Parameter Estimation in a Physiologically-Based Pharmacokinetic Model of Ethanol." Biomed Signal Process Control **2**(2): 97-110.

Pruim, R. H. R., M. Mennes, D. van Rooij, A. Llera, J. K. Buitelaar and C. F. Beckmann (2015). "ICA-AROMA: a robust ICA-based strategy for removing motion artifacts from fMRI data." Neuroimage **112**: 267-277.

Rolls, E. T., C. C. Huang, C. P. Lin, J. Feng and M. Joliot (2020). "Automated anatomical labelling atlas 3." Neuroimage **206**: 116189.

Seo, D., C. M. Lacadie, K. Tuit, K. I. Hong, R. T. Constable and R. Sinha (2013). "Disrupted ventromedial prefrontal function, alcohol craving, and subsequent relapse risk." JAMA Psychiatry **70**(7): 727-739.

Sjoerds, Z., S. de Wit, W. van den Brink, T. W. Robbins, A. T. Beekman, B. W. Penninx and D. J. Veltman (2013). "Behavioral and neuroimaging evidence for overreliance on habit learning in alcohol-dependent patients." Transl Psychiatry **3**: e337.

Tian, Y., D. S. Margulies, M. Breakspear and A. Zalesky (2020). "Topographic organization of the human subcortex unveiled with functional connectivity gradients." Nat Neurosci **23**(11): 1421-1432.

Voon, V., E. Grodin, A. Mandali, L. Morris, N. Donamayor, K. Weidacker, L. Kwako, D. Goldman, G. F. Koob and R. Momenan (2020). "Addictions NeuroImaging Assessment (ANIA): Towards an integrative framework for alcohol use disorder." Neurosci Biobehav Rev **113**: 492-506.

Wiers, C. E., C. Stelzel, T. E. Gladwin, S. Q. Park, S. Pawelczack, C. K. Gawron, H. Stuke, A. Heinz, R. W. Wiers, M. Rinck, J. Lindenmeyer, H. Walter and F. Bermpohl (2015). "Effects of cognitive bias modification training on neural alcohol cue reactivity in alcohol dependence." Am J Psychiatry **172**(4): 335-343.
