# Supplementary material for: Brain response in heavy drinkers during cross-commodity alcohol and money discounting with potentially real rewards: A preliminary study
Source: Drug Alcohol Depend Rep. 2023 Jul 6;8:100175. doi: 10.1016/j.dadr.2023.100175 (PMC10518510; doi:10.1016/j.dadr.2023.100175)
Supplement: Supplementary file 3 [file mmc3.docx]

| Table S1  Principal Component Analysis of Alcohol-related and Personality Risk Factors (*n*=24) | | | |
| --- | --- | --- | --- |
|  | Component | | |
|  | Drinking problems/ Emotionality | | Alcohol Consumption |
| Drinks per week | .09 | .99* | |
| Heavy drinking days per week | .01 | .46* | |
| Drinks per drinking day | .26 | .80* | |
| DSM-IV criteria, lifetime | .54* | .19 | |
| AUDIT | .76* | .17 | |
| CES-D | .96* | .19 | |
| AISS-Novelty | .22 | −.38 | |
| AISS-Intensity | −.19 | −.15 | |
| SUPPSP-Negative Urgency | .46* | −.11 | |
| SUPPSP-Lack of Perseverance | −.00 | −.19 | |
| SUPPSP-Lack of Premeditation | .12 | .00 | |
| SUPPSP-Sensation Seeking | −.06 | −.28 | |
| SUPPSP-Positive Urgency | .56* | −.09 | |

*High factor loadings (>0.4) characterize component labels

Varimax rotated solutions shown.

DSM=Diagnostic and Statistical Manual of Mental Disorders; AUDIT=Alcohol Use Disorders Identification Test; CES-D=Center for Epidemiologic Studies Depression Scale; AISS=Arnett Inventory of Sensation Seeking; SUPPSP=(Short) Urgency, Premeditation, Perseverance, Sensation seeking, Positive urgency impulsivity scale.

| Table S2  Personality Descriptive Statistics (*n*=24) | | | |
| --- | --- | --- | --- |
|  | Mean ±SD | | Range |
| CES-D | 12.2 ±11.2 | 1 – 46 | |
| AISS-Novelty | 29.0 ±3.2 | 23 – 34 | |
| AISS-Intensity | 26.3 ±4.1 | 18 – 35 | |
| SUPPSP-Negative Urgency | 9.8 ±3.2 | 4 – 15 | |
| SUPPSP-Lack of Perseverance | 6.8 ±1.9 | 4 – 11 | |
| SUPPSP-Lack of Premeditation | 7.1 ±2.4 | 4 – 11 | |
| SUPPSP-Sensation Seeking | 11.4 ±2.9 | 6 – 16 | |
| SUPPSP-Positive Urgency | 8.5 ±3.2 | 4 – 16 | |

DSM=Diagnostic and Statistical Manual of Mental Disorders; AUDIT=Alcohol Use Disorders Identification Test; CES-D=Center for Epidemiologic Studies Depression Scale; AISS=Arnett Inventory of Sensation Seeking; SUPPSP=(Short) Urgency, Premeditation, Perseverance, Sensation seeking, Positive urgency impulsivity scale.

Table S3. Drug use history by drug category.

| Drug type | Lifetime use (%) | Lifetime use >10x (%) | Recent use^a^ (%) |
| --- | --- | --- | --- |
| THC | 96 | 71 | 55 |
| Stimulants | 38 | 21 | 14 |
| Sedatives | 50 | 21 | 5 |
| Other | 54 | 29 | 0 |

THC: marijuana/cannabis derived substances; Stimulants: amphetamines, cocaine; Sedatives: benzodiazepines, opiates, barbiturates, sedative hypnotics; Other: hallucinogens (LSD, psilocybin, mescaline), inhalants, nitrous oxide, MDA, MDMA, THC analogs.

^a^Any use within the past 6 months.
